# Epalrestat Alleviates Reactive Oxygen Species and Endoplasmic Reticulum Stress by Maintaining Glycosylation in IMS32 Schwann Cells Under Exposure to Galactosemic Conditions

**DOI:** 10.3390/ijms26041529

**Published:** 2025-02-12

**Authors:** Hideji Yako, Naoko Niimi, Shizuka Takaku, Junji Yamauchi, Kazunori Sango

**Affiliations:** 1 Diabetic Neuropathy Project, Tokyo Metropolitan Institute of Medical Science, Tokyo 156-8506, Japan; niimi-nk@igakuken.or.jp (N.N.); takaku-sz@igakuken.or.jp (S.T.); yamauchi@toyaku.ac.jp (J.Y.); 2 Laboratory of Molecular Neuroscience and Neurology, Tokyo University of Pharmacy and Life Sciences, Tokyo 192-0392, Japan; 3 Laboratory of Molecular Pharmacology, National Research Institute for Child Health and Development, Tokyo 157-8535, Japan

**Keywords:** aldose reductase, epalrestat, endoplasmic reticulum stress, glycosylation, high-galactose conditions, Schwann cells

## Abstract

Aldose reductase (AR), a rate-limiting enzyme in the polyol pathway, mediates the conversion of several substrates, including glucose and galactose. In rodents, galactosemia induced by galactose feeding has been shown to develop peripheral nerve lesions resembling diabetic peripheral neuropathy. However, the mechanisms by which AR-mediated responses elicited Schwan cell lesions under galactosemic conditions remain unresolved. To investigate this, we examined the mechanism of high-galactose-induced damage mediated by AR using AR inhibitors such as ranirestat and epalrestat. The exposure of IMS32 Schwann cells under high-galactose conditions led to galactitol accumulation, the increased production of reactive oxygen species (ROS), endoplasmic reticulum (ER) stress, impaired mitochondrial morphology and membrane potential, decreased glycolysis, and aberrant glycosylation. Under these experimental conditions, ranirestat inhibited intracellular galactitol in a dose-dependent manner, whereas epalrestat failed to inhibit it. Interestingly, even at low concentrations where epalrestat did not inhibit AR activity, it prevented increased ROS production, ER stress, decreased glycolysis, and aberrant RCA120-binding glycosylation; however, no effect of ranirestat on the glycosylation was observed. Epalrestat and ranirestat did not recover mitochondrial morphology. These findings suggest that ER stress is induced by aberrant glycosylation under galactosemic conditions and that epalrestat may be effective in maintaining proper glycosylation in Schwann cells in these conditions.

## 1. Introduction

The polyol pathway, a collateral pathway in glycolysis, converts glucose to sorbitol via the enzyme aldose reductase (AR) and subsequently metabolizes sorbitol to fructose through sorbitol dehydrogenase (SDH) [1]. The accumulation of metabolites from the polyol pathway is considered a major pathogenic factor in diabetic peripheral neuropathy (DPN) [2,3]. DPN, a chronic complication of diabetes mellitus, presents with distal symmetrical neurological symptoms with axonal-length-dependent damage and aberrant myelin sheath abnormalities. These are driven by hyperglycemia-induced oxidative stress [4,5], osmotic stress [6], endoplasmic reticulum (ER) stress [7,8,9], and the reduced production and/or activity of neurotrophic factors [10,11].

In addition to converting glucose to sorbitol, AR catalyzes the reduction of galactose to galactitol. Galactosemia induced by galactose load leads to galactitol accumulation in Schwann cells [12] and peripheral nerves [13,14,15,16], which is associated with increased oxidative and osmotic stress [17,18,19], as well as a reduced production and/or bioactivity of neurotrophic factors like brain-derived neurotrophic factor [20], neurotrophin-3 [21], and ciliary neurotrophic factor [14] in peripheral nerves. These changes can lead to decreased motor and sensory nerve conduction velocities and myelin sheath abnormalities [14,17,18,20,21,22]. Galactosemic neuropathy is worsened in human AR-transgenic mice [17] and alleviated by AR inhibitors [13,14,15,16], suggesting that AR plays a harmful role in its development and progression. AR is highly expressed in Schwann cells in the peripheral nervous system [23]. However, the molecular mechanism by which AR mediates galactose-induced injury in Schwann cells remains unclear.

AR reduces glucose and galactose using nicotinamide adenine dinucleotide phosphate (NADPH) as a cofactor. Sorbitol is then converted into fructose by SDH in an NAD-dependent reaction, whereas galactitol is not further metabolized. Besides AR-mediated reduction, galactose is also metabolized through the Leloir pathway, a key catabolic pathway that converts galactose to glucose-6-phosphate, linking it to glycolysis [24]. These metabolic pathways imply (1) no significant effect on SDH and NAD in the polyol pathway (i.e., only AR is involved in the reaction) and (2) an enhancement in glycolytic flux under galactosemic conditions. Additionally, AR has a much lower Km value for galactose than for glucose [25], suggesting that galactose may serve as a more effective substrate than glucose for investigating AR-related pathogenic mechanisms in Schwann cells.

Epalrestat is the only AR inhibitor available for clinical use in Japan and a few other Asian countries. Long-term treatment with epalrestat is useful for ameliorating the progression and restoration of the DPN symptoms such as sorbitol accumulation and nerve conduction velocity, in particular, in patients with good glycemic control. However, patients with poor glycemic control are not effective in progressing and restoring the DPN symptoms. Ranirestat was more effective than epalrestat in suppressing AR in cells, animals, and patients under hyperglycemia [26,27] and was discontinued in development. While epalrestat has a beneficial role in ameliorating phosphomannomutase 2-congenital disorder of glycosylation (PMM2-CDG) [28,29], the efficacy of epalrestat is undergoing assessment in the clinical trial of PMM2-CDG (NCT04925960). In addition to AR, also known as aldo-keto reductase (AKR)1B, epalrestat has been shown to inhibit the activity of AKR1B10, a member of the AKR superfamily. AKR1B10 is overexpressed in various cancers and is considered a potential therapeutic target. In drug-resistant cancers, the combination of epalrestat and chemotherapy results in cell death [30,31]. In recent years, the efficacy of epalrestat against cultured cells and rodents has been reported in cancer [30], liver diseases [32,33], and neuronal diseases [34,35].

This study aims to elucidate the pathological roles of AR and its mediated responses in Schwann cell damage under galactosemic conditions, as well as to assess the potential efficacy of the two kinds of AR inhibitors, ranirestat and epalrestat, in mitigating these lesions.

## 2. Results

### 2.1. Ranirestat Prevented Galactitol Accumulation in IMS32 Schwann Cells Under Galactosemic Conditions

**Figure 1:**
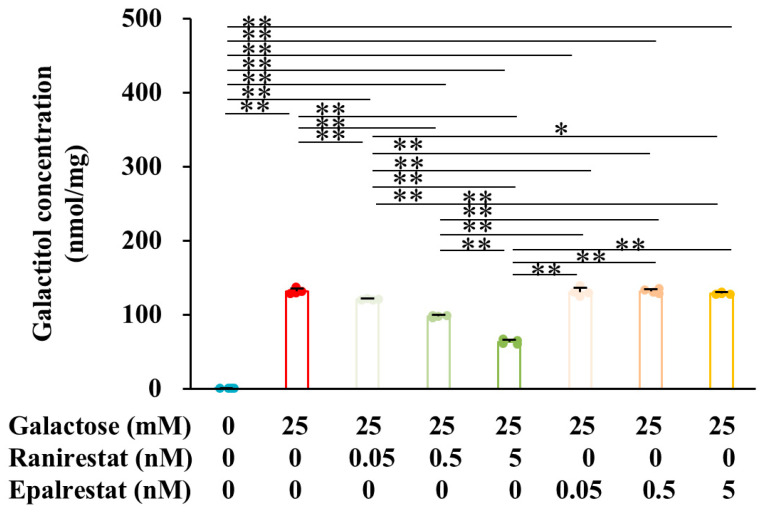
Ranirestat, but not epalrestat, inhibited galactitol accumulation in IMS32 cells exposed to galactosemic conditions. Galactitol levels in IMS32 cells under control (blue) and high-galactose (red) conditions, as well as in the presence of 0.05, 0.5, and 5 nM ranirestat (green) and epalrestat (yellow), are shown. Values represent the mean + SD from four experiments, with individual values depicted as circles. * *p* < 0.05, ** *p* < 0.01.

Since AR catalyzes the NADPH-dependent conversion of galactose to galactitol, we assessed intracellular galactitol concentration, AR expression, and the NADP/NADPH ratio in IMS32 Schwann cells under normal (galactose-free) and galactosemic (25 mM galactose) conditions. Treatment with 50 and 500 nM ranirestat, but not 500 nM epalrestat, under high-glucose conditions has been reported to inhibit intracellular sorbitol levels in human umbilical vein endothelial cells [26]. The IC50 values of ranirestat and epalrestat are estimated to be approximately 10 and 20 nM, respectively [6,36]. Therefore, we further investigated the inhibitory efficacy of ranirestat and epalrestat on galactitol accumulation in IMS32 cells under high-galactose conditions. As anticipated, the galactose load significantly increased intracellular galactitol levels (Figure 1). Ranirestat inhibited galactitol accumulation in a dose-dependent manner (0.05 nM < 0.5 nM < 5 nM), whereas epalrestat did not reduce galactitol levels even at the highest concentration (5 nM) tested (Figure 1). These results suggest that ranirestat is more effective than epalrestat in inhibiting AR under galactosemic conditions. We further examined the therapeutic efficacy of ranirestat and epalrestat on high-galactose-induced damages using the highest concentration of our experiment (5 nM).

**Figure 2:**
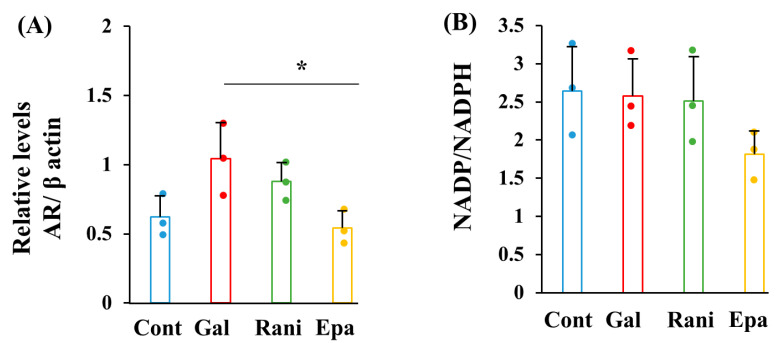
AR inhibitors suppressed AR expression under high-galactose conditions. AR expression (**A**) and the NADP/NADPH ratio (**B**) were assessed in the following conditions: control (Cont; blue), high galactose (Gal; red), high galactose with 5 nM ranirestat (Rani; green), and high galactose with 5 nM epalrestat (Epa; yellow). The actual blots for AR and β actin (**A**) are shown in Figure S4. Values represent the mean + SD from three experiments, with individual values depicted as circles. * *p* < 0.05.

AR expression, measured by Western blotting, tended to increase in response to the galactose load, but it was suppressed with the supplementation of ranirestat and, more markedly, epalrestat (Figure 2A). However, the intracellular NADPH/NADP ratio remained unchanged in response to the galactose load, regardless of the presence of AR inhibitors (Figure 2B).

### 2.2. Ranirestat and Epalrestat Prevented ROS Production Under Galactosemic Conditions

**Figure 3:**
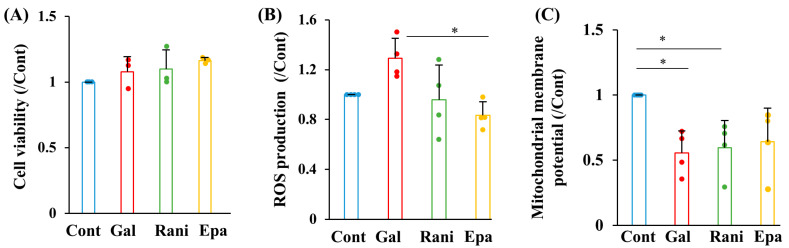
AR inhibitors attenuated ROS production under galactosemic conditions. Cell viability (**A**), ROS production (**B**), and mitochondrial membrane potential (**C**) were measured under the following conditions: control (Cont; blue), high galactose (Gal; red), high galactose with 5 nM ranirestat (Rani; green), and high galactose with 5 nM epalrestat (Epa; yellow). Values represent the mean + SD from three experiments for cell viability (**A**) and four experiments for ROS production and mitochondrial membrane potential (**B**,**C**), with individual values depicted as circles. * *p* < 0.05.

To further investigate the mechanisms of galactose toxicity in Schwann cells and the therapeutic efficacy of ranirestat and epalrestat, we assessed IMS32 cell viability, reactive oxygen species (ROS) production, and mitochondrial membrane potential using MTS assay, DCFH-DA, an ROS indicator, and JC-10, a mitochondrial membrane potential indicator, respectively. Galactose exposure, with or without ranirestat or epalrestat, had no significant effect on cell viability (Figure 3A). The galactose load tended to increase ROS production, which was prevented by supplementation with both ranirestat and epalrestat; the latter showed greater efficacy (Figure 3B). However, neither AR inhibitors prevented the galactose-induced reduction in mitochondrial membrane potential (Figure 3C). These findings suggest that ranirestat and epalrestat protected IMS32 cells from galactose-induced ROS production but not mitochondrial dysfunction.

Heme oxygenase-1 (HO-1), an antioxidant enzyme, has been shown to be upregulated at both mRNA and protein levels by epalrestat in neurons and Schwann cells [37]. Superoxide dismutase 2 (SOD2), a mitochondrial antioxidant enzyme, also plays a protective role against ROS and DPN [38]. However, Western blotting revealed that HO-1 and SOD2 levels in IMS32 cells exposed to galactosemic conditions were unaltered, with levels even slightly decreased by AR inhibitor treatment (Figure S1). These data suggest that neither ranirestat nor epalrestat contribute to the upregulation of antioxidant enzymes in response to galactose-induced ROS production.

### 2.3. Ranirestat and Epalrestat Ameliorated Glycolytic Flux Under Galactosemic Conditions

**Figure 4:**
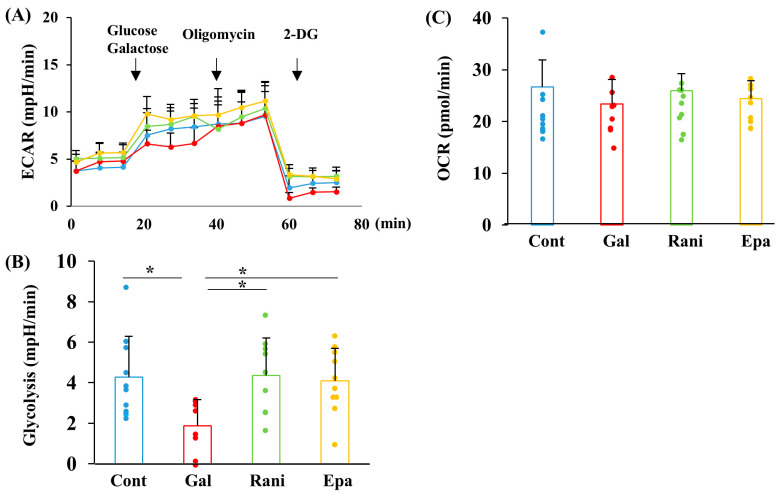
AR inhibitors ameliorated the galactose-induced impairment of glycolysis. The representative data for extracellular acidification rate (ECAR) (**A**), glycolysis flux (**B**), and oxygen consumption rate (OCR) (**C**) from the XF Glycolysis Stress Test in IMS32 cells under the following conditions are shown: control (Cont; blue), high galactose (Gal; red), high galactose with 5 nM ranirestat (Rani; green), and high galactose with 5 nM epalrestat (Epa; yellow). Glycolysis was calculated based on the differences in ECAR between glucose and oligomycin injections. OCR values at 21 min after measurement are presented. Values represent the mean + SD from 8 to 10 experiments, with individual values depicted as circles. * *p* < 0.05.

Glucose-6-phosphate is produced from galactose through the Leloir pathway and links to glycolysis. Therefore, exposure to galactose, as well as high glucose, is expected to alter glycolytic flux. As anticipated, the galactose load inhibited glycolytic flux, as measured by the extracellular acidification rate (ECAR) using the Extracellular Flux Analyzer, which was prevented by supplementation with ranirestat and epalrestat (Figure 4A,B). In contrast to glycolysis, mitochondrial respiration (oxygen consumption rate; OCR), assessed by the Extracellular Flux Analyzer, was unaffected by galactose load, regardless of the presence or absence of the AR inhibitors (Figure 4C). These results suggest that ranirestat and epalrestat help restore galactose-induced disturbances in glycolysis.

### 2.4. Ranirestat and Epalrestat Protected IMS32 Cells from Galactose-Induced ER Stress

**Figure 5:**
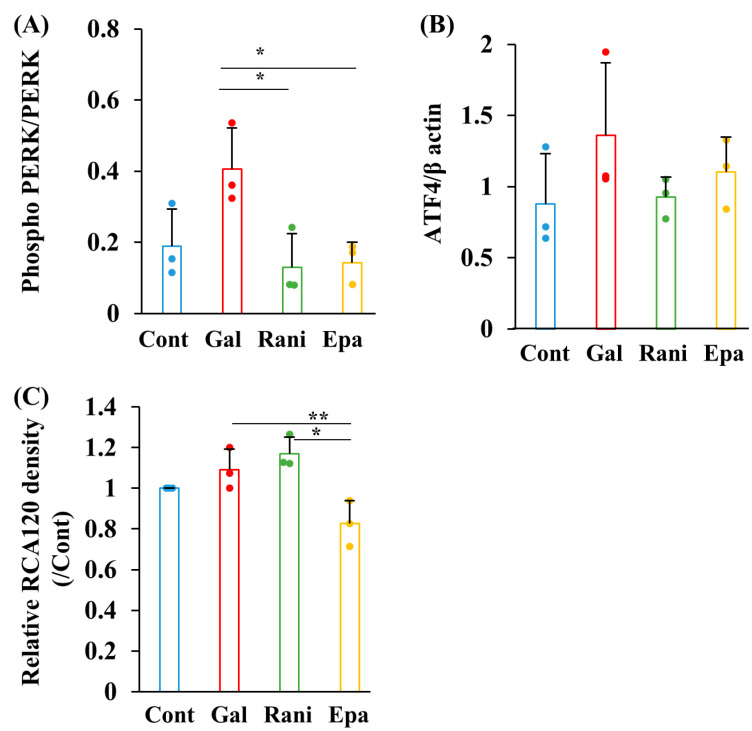
AR inhibitors attenuated ER stress under high-galactose conditions. Levels of ER stress markers, phosphorylated PERK (**A**) and ATF4 (**B**), and the galactose-binding lectin RCA120 (**C**) were assessed by Western blotting and dot blot analysis. Bar graphs show the levels of these factors in IMS32 cells under the following conditions: control (Cont; blue), high galactose (Gal; red), high galactose with 5 nM ranirestat (Rani; green), and high galactose with 5 nM epalrestat (Epa; yellow). Actual blots for phosphorylated PERK, PERK, ATF4, RCA120, and β actin are shown in Figure S5. Values represent the mean + SD from three experiments, with individual values depicted as circles. * *p* < 0.05, ** *p* < 0.01.

ER stress, triggered by the accumulation of unfolded proteins, has been associated with AR activity in the lens of galactosemic rats [39] and the liver of patients with alcoholic liver disease [33]. To investigate the impact of AR inhibitors on ER-stress-related proteins, we assessed the expression of phosphorylated PERK and ATF4 by Western blotting. The galactose-induced upregulation of phosphorylated PERK and ATF4 was reversed by supplementation with ranirestat and epalrestat (Figure 5A,B), suggesting that these AR inhibitors protect Schwann cells from ER stress induced by galactosemic conditions. UDP-galactose, a substrate necessary for glycosylation, is produced through the Leloir pathway. Aberrant glycosylation can lead to ER stress, so RCA120, a galactose-binding lectin, was used to assess protein glycosylation. RCA120 levels, measured by dot blot, tended to increase under high-galactose conditions compared to the control. Notably, epalrestat, but not ranirestat, significantly reduced RCA120 levels under galactosemic conditions (Figure 5C). We further examined whether high-dose epalrestat could maintain glycosylation at concentrations that inhibited AR activity. Galactosemia-induced ER stress was attenuated by epalrestat in a dose-dependent manner (Figure S2). These results suggest that epalrestat and ranirestat reduce ER stress through distinct mechanisms under high-galactose conditions.

### 2.5. Galactose Load Resulted in Morphological Changes in IMS32 Cells

**Figure 6:**
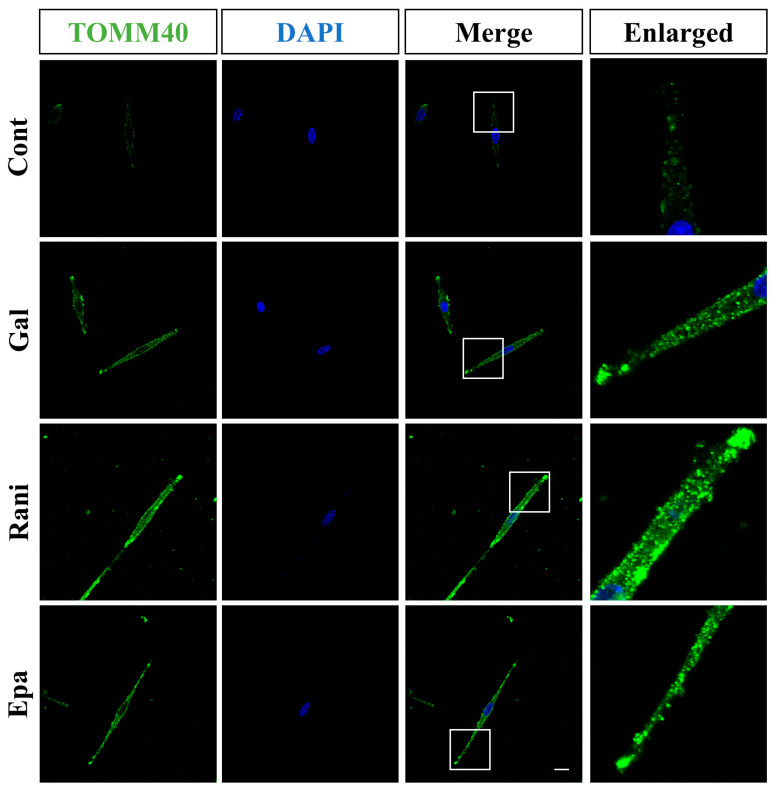
TOMM40 signals increased and aggregated under high-galactose conditions. Representative immunostaining images of TOMM40 (green) and nuclear staining of DAPI (blue) in IMS32 cells under the following conditions are shown: control (Cont), high galactose (Gal), high galactose with 5 nM ranirestat (Rani), and high galactose with 5 nM epalrestat (Epa). Enlarged images of the white-boxed regions in the merged images are also shown. Scale bar: 10 μm.

We examined the morphology of mitochondria and ER in IMS32 cells under high-galactose conditions using immunocytochemistry. Signals of TOMM40, a mitochondrial outer membrane marker, were found to increase and aggregate under these conditions and remained unchanged even after supplementation with ranirestat and epalrestat (Figure 6). To assess ER morphology, we used the KDEL antigen, an ER-resident Lys-Asp-Glu-Leu oligopeptide sequence commonly associated with the retention of ER proteins. Neither galactose load nor supplementation with ranirestat and epalrestat altered the localization of KDEL signals in IMS 32 cells (Figure S3). These results suggest that galactosemic conditions resulted in changes in mitochondrial morphology as observed using a confocal laser scan microscope.

## 3. Discussion

This study demonstrates galactose-induced Schwann cell damage mediated by AR and highlights the significant role of epalrestat in maintaining glycosylation in IMS32 Schwann cells under galactosemic conditions. The experimental concentration of epalrestat (5 nM) did not inhibit intracellular galactitol accumulation, suggesting no effect on AR activity, yet it alleviated ER stress and ROS production. This study also indicates that ranirestat and epalrestat reduce galactose-induced ROS production and ER stress via a distinct pathway.

Hyperglycemia triggers oxidative stress [4,5], osmotic stress [6], and ER stress [7,8,9], all contributing factors to DPN. Similarly, galactosemic conditions appear to promote peripheral neuropathy through these stress pathways [17,18]. In Schwann cells of galactose-fed rats, amorphous and granular protein structures were observed within the dilated ER cisternae [13]. Galactose feeding has also been shown to induce cataracts via ER stress, which was ameliorated by AR inhibitors [39]. Together with our findings, these results suggest that galactosemia induces ER stress in Schwann cells via AR-mediated response.

Galactitol accumulation results from AR-mediated galactose reduction, while sorbitol accumulates due to SDH inhibition. However, neither SDH-deficient mice [40] nor an SDH inhibitor [41] improved nerve function in STZ-diabetic rodents. Similarly, AR inhibitors but not SDH inhibitors alleviated cataract development in galactose-fed and STZ-induced diabetic rats [42]. These findings emphasize that AR activity and polyol accumulation substantially contribute to the development of DPN and diabetic retinopathy.

Exposure to galactosemic conditions increased ROS production and impaired mitochondrial membrane potential; yet, it did not affect mitochondrial respiration in IMS32 cells. Ranirestat and epalrestat both reduced ROS production but did not prevent the decline in mitochondrial membrane potential, suggesting that these AR inhibitors lack direct antioxidant activity. Mitochondrial damages and galactitol accumulation were observed in the sciatic nerves of galactose-fed animals treated with AR inhibitors [13]. Despite ranirestat’s inhibition of galactitol accumulation, it was insufficient in preventing decreases in mitochondrial membrane potential, possibly due to osmotic stress from elevated intracellular galactitol. We also observed increased and aggregated TOMM40 under galactosemic conditions and it remained unaltered with ranirestat and epalrestat supplementation. These findings indicate that TOMM40 overexpression induced by high-galactose load may contribute to mitochondrial dysfunction. However, there are the discrepancy findings regarding the relationship between TOMM40 expression and mitochondrial function. TOMM40 overexpression has been shown to enhance mitochondrial function, increasing activities of oxidative phosphorylation complexes, cellular ATP levels, and mitochondrial membrane potential [43]. This overexpression also protects against Aβ-induced mitochondrial dysfunction [43]. However, elevated TOMM40 RNA levels in the brains of Alzheimer’s disease patients have been associated with decreased mitochondrial DNA copy number and membrane potential under oxidative stress [44]. Furthermore, mitochondrial degenerative changes such as enlarged mitochondria are developed by galactose feeding and are not rescued by an AR inhibitor [13]. These results suggest that elevated TOMM40 signals induced by galactosemia may impair the mitochondrial membrane potential. In addition to mitochondria, the shape of ER in IMS32 cells was also assessed. ER forms a sheet and tubule structure, which dynamically change [45]. Using KDEL, an ER marker, we found no significant changes in ER signals under galactosemic conditions as observed using laser scanning microscopy. Whether ER stress impacts ER morphology remains unclear. Further study is needed to elucidate the relationship between ER stress and shape in IMS32 cells under galactosemic conditions.

In the Leloir pathway, UDP-galactose, a glycosylation substrate, is produced, and its aberrant generation may induce ER stress. ROS production can also induce ER stress. Exposure to galactosemic conditions led to increased ROS, ER stress, and aberrant RCA120-binding glycosylation, as well as reduced glycolytic flux in IMS32 cells. Both ranirestat and epalrestat reversed galactose-induced ROS production, ER stress, and glycolytic disturbances; however, only epalrestat corrected the aberrant glycosylation. Ranirestat, but not epalrestat, significantly reduced galactitol accumulation under high-galactose conditions. Higher doses of epalrestat that inhibited AR activity also restored glycosylation. Glycolytic flux is suppressed by ROS production induced by hyperglycemia as well as galactosemia [46,47,48]. These results suggest that galactitol accumulation mediated by AR contributes to ROS production and ER stress, which in turn suppress glycolysis. In addition to AR inhibition, epalrestat appears to support glycosylation, though the specific mechanism remains unclear.

Phosphomannomutase 2 (PMM2), which converts mannose-6-phosphate to mannose-1-phosphate, plays a critical role in glycosylation. PMM2 deficiency is the most common congenital disorder of glycosylation (PMM2-CDG). Epalrestat and siRNA-induced suppression of AR, but not ranirestat, improved PMM2 activity via AR-inhibition-induced increases in glucose-1,6-diphosphate in PMM2 mutant worms and patient-derived fibroblasts [28,29]. These studies identified the terminal carboxylic moiety of epalrestat as essential for improving PMM2 activity and glycosylation [28,29]. Epalrestat and ranirestat are classified as a carboxylic acid derivative and a succinimide compound, respectively, and both interact with the active site residues of AR [49]. Epalrestat binds to Trp 20 and Trp 111 in AR through hydrogen bonds involving amino acid side-chain and π-π interactions, respectively. In contrast, ranirestat binds to Trp 48 and Trp 111 in AR via hydrogen bonds involving amino acid side chains [49]. Epalrestat has been shown to cross the blood–brain-barrier [44] and activate NRF2 [37], whereas ranirestat remains undetermined. These differences may lead to the efficacy of epalrestat, but not ranirestat, on RCA120-related glycosylation in IMS32 cells under galactosemic conditions. The concept of AR differential inhibitors (ARDI) has been proposed [50], where specific substrates selectively inhibit AR activity. The inhibitory effects of epalrestat and ortho-fluoro para-bromo compounds, including ranirestat, were negligible or slight on substrates like L-idose and hydroxynonenal [51], suggesting minimal substrate specificity differences. However, our study shows that AR inhibition differentially affects glycosylation. Further research is needed to elucidate AR’s involvement in glycosylation.

Hereditary galactosemia, such as galactose-1-phosphate uridylyltransferase deficiency, presents symptoms after milk intake, including failure to thrive, diarrhea, or other less common complications such as renal tubule dysfunction or cataract [52]. The mechanisms underlying these symptoms are hypothesized to involve increased ER stress, elevated osmotic stress, upregulated oxidative stress, or aberrant glycosylation [39,53,54,55]. Although peripheral neuropathy is not typically reported in patients with hereditary galactosemia, animal studies have shown that galactose feeding develops peripheral neuropathy [13,14,18,20,21]. These findings suggest that epalrestat may represent a potential therapeutic target for hereditary galactosemia.

**Figure 7:**
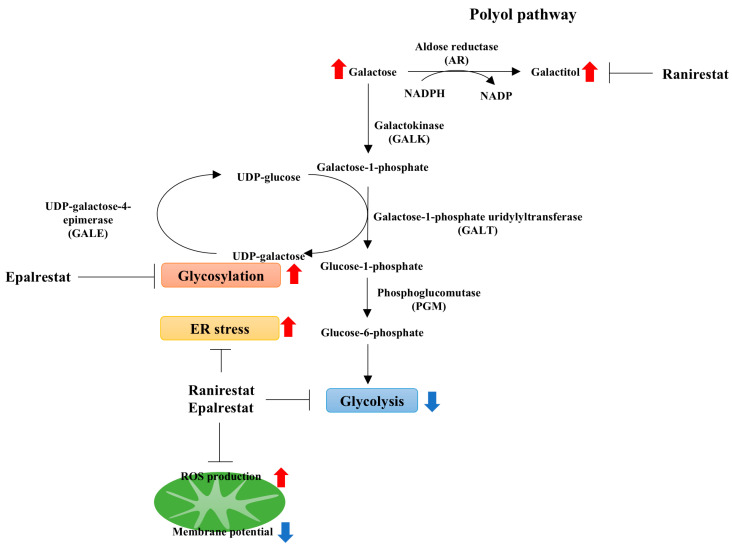
The schematic representation of metabolic changes in IMS32 cells under galactosemic conditions. IMS32 cells exposed to galactosemic conditions exhibited galactitol accumulation, abnormal glycosylation, increased ROS production and ER stress, reduced glycolysis, and impaired mitochondrial membrane potential. Treatment with epalrestat improved glycosylation, ROS production, ER stress, and glycolysis, whereas ranirestat restored galactitol accumulation, ROS production, ER stress, and glycolysis. However, AR inhibitors were unable to recover mitochondrial membrane potential. Blue arrows indicate decreases, while red arrows indicate increases.

Galactose-induced Schwann cell damage may result from various mechanisms, including galactitol accumulation, aberrant RCA120-binding glycosylation, increased ROS production, ER stress, decreased glycolysis, and impaired mitochondrial membrane potential (Figure 7). The precise mechanisms underlying the decline in mitochondrial membrane potential and the high-resolution morphology of ER using electron microscopy remain to be clarified. This study also demonstrates the potential utility of epalrestat in maintaining glycosylation under galactosemic conditions and the greater inhibitory efficacy of ranirestat compared to epalrestat. However, further studies are necessary to understand how epalrestat prevents aberrant glycosylation in Schwann cells under high-galactose conditions.

## 4. Materials and Methods

### 4.1. Cell Culture

Spontaneously immortalized adult mouse Schwann (IMS32) cells were established and maintained by our laboratory (previously known as the ALS/neuropathy project) [56]. IMS32 cells were seeded at an approximate density of 1.8 × 10^4^ cells/cm^2^ in 96-well plates, as well as on 60 mm and 100 mm dishes and cover glass (Matsunami Glass Industry, Osaka, Japan), and then incubated for 3 days in Dulbecco’s Modified Eagle’s medium (DMEM; Nakarai Tesque., Kyoto, Japan) supplemented with 5% fetal bovine serum (FBS; Thermo Fisher Scientific Inc., Waltham, MA, USA) and an Antibiotic–Antimycotic Mixed Solution (100 units/mL of penicillin, 100 μg/mL of streptomycin; Nakarai Tesque). IMS32 cells were subsequently maintained in DMEM containing either 0 or 25 mM galactose (FUJIFILM Wako Pure Chemical Corp., Osaka, Japan), with or without ranirestat or epalrestat at concentrations of 0.05, 0.5, 5, 500, or 5000 nM (provided by Sumitomo Pharma Co., Ltd., Osaka, Japan) for 1 or 2 days.

### 4.2. Measurement of Galactitol in IMS32 Cells Using Liquid Chromatography Coupled with Tandem Mass Spectrometry (LC/MS/MS)

After the incubation period described under “Cell culture”, intracellular galactitol concentrations were measured using an LC/MS/MS system at Sumika Chemical Analysis Service, Ltd. (Osaka, Japan), in collaboration with Sumitomo Pharma Co., Ltd. (Osaka, Japan) [12].

### 4.3. Measurement of Cell Viability

IMS32 cell viability was assessed using the CellTiter 96 AQueous One Solution Cell Proliferation Assay kit (Promega, Madison, WI, USA) following the manufacturer’s instructions. Briefly, AQueous One Solution was added to each well, and cells were incubated at 37 °C for 2 h. Absorbance at 490 nm was measured using a plate reader (Valioskan Flash, Thermo Fisher Scientific Inc.).

### 4.4. Measurement of ROS Production

ROS production in seeded IMS32 cells on a 96-well black plate exposed under such conditions described by Cell culture was evaluated using the ROS Assay Kit Highly Sensitive DCFA-DA (Dojindo, Kumamoto, Japan) in accordance with the manufacturer’s instructions. Highly Sensitive DCFH-DA diluted by loading buffer (1:1000) was added to each well, and cells were incubated for 30 min at 37 °C. After washing with PBS, fluorescence intensity (excitation: 490 nm, emission: 525 nm) was measured using a plate reader (Valioskan Flash).

### 4.5. Measurement of Mitochondrial Membrane Potential

The mitochondrial membrane potential in IMS32 cells was determined using the JC-10 Mitochondrial Membrane Potential Assay Kit-Microplate (Abcam, Cambridge, UK) as per the manufacturer’s instructions. Briefly, JC-10 dye-loading solution was added to each well, and cells were incubated for 30 min at room temperature. Following the addition of Buffer B, fluorescence intensities (excitation: 490/525 nm, emission: 540/590 nm) were measured using a plate reader (Valioskan Flash).

### 4.6. Measurement of NADP and NADPH Levels

NADP and NADPH levels in IMS32 cells were estimated using the NADP and NADPH Assay Kit-WST (Dojindo), following the manufacturer’s instructions. Briefly, samples prepared by extraction buffer and filtration tube incubation were added to each well. Working solution was added to each well and then incubated for 60 min at 37 °C. The absorbance at 490 nm was obtained using a plate reader (Valioskan Flash), and the NADP/NADPH ratio was calculated.

### 4.7. Western Blotting

Western blot analysis was performed as previously described [46,47], with slight modifications. Briefly, cells were lysed in RIPA buffer (FUJIFILM Wako Pure Chemical Corp.) with protease inhibitor cocktails (Takara Bio Inc., Kusatsu, Japan). Cell lysates were sonicated using a Handy Sonic (TOMY SEIKO Co., Ltd., Tokyo, Japan), incubated with NuPAGE LDL sample buffer and reducing agent at 70 °C (Thermo Fisher Scientific Inc.), and loaded onto 4–12% NuPAGE Bis-Tris Mini Gel (Thermo Fisher Inc.). Proteins were transferred onto polyvinylidene fluoride membranes (EMD Millipore, Burlington, MA, USA) using an electroblotter (Bio-Rad, Hercules, CA, USA) for 1 h at 4 °C. After washing with TBST, membranes were immersed in blocking buffer (10 mM Tris, pH 7.4, 150 mM NaCl, 5% bovine serum albumin, and 0.1% Tween 20) for 1 h, followed by washing with TBST. Membranes were incubated overnight at 4 °C with primary antibodies: mouse anti-β-actin monoclonal antibody (1:4000; Sigma-Aldrich Co. LCC., St Louis, MO, USA), goat anti-AR polyclonal antibody (Santa Cruz Biotechnology, Inc., Santa Cruz, TX, USA), rabbit anti-Phospho PERK monoclonal antibody (1:500; Cell Signaling Technology, Beverly, MA, USA), rabbit anti-PERK monoclonal antibody (1:1000; Cell Signaling Technology), and rabbit anti-ATF4 polyclonal antibody (1:1000; Proteintech Group, Inc., Rosemont, IL, USA). After rinsing with TBST, immunoblotting was performed with horseradish peroxidase (HRP)-conjugated secondly antibodies (anti-mouse IgG, anti-goat IgG, and anti-rabbit IgG, 1:2000, MBL Corp., Ltd., Nagoya, Japan) for 1 h at room temperature. Bands were visualized using the ECL plus Western Blotting Detection Kit (GE Healthcare Technologies Inc., Chicago, IL, USA), and images were obtained with the EZ capture Ⅱchemiluminescence imaging system (ATTO, Tokyo, Japan). Signal intensity was quantified using Image J, and the relative intensity of each protein was normalized to β-actin.

### 4.8. Measurement of Glycosylation (Dot Blot)

An amount of 5 μL of cell lysates (1 μg/μL) prepared for Western blotting was applied to nitrocellulose membranes (Amersham International plc, Amersham, UK). After air drying, membranes were immersed in the blocking buffer for 1 h and then washed with TBST. Membranes were incubated overnight at 4 °C with RCA120-HRP, a galactose-binding lectin (1:100, diluted by blocking buffer, J OIL MILLS, Inc., Tokyo, Japan). After rinsing with TBST, blots were visualized with the ECL plus Western Blotting Detection Kit. Images were obtained using the EZ capture II system, and signal intensity was quantified using Image J (version 1.54g).

### 4.9. Measurement of Extracellular Acidification Rate (ECAR) and Oxygen Consumption Rate (OCR)

ECAR and OCR measurements were performed using the XFe96 Extracellular Flux Analyzer (Agilent Technologies, Santa Clara, CA, USA) as previously described [46]. For ECAR and OCR analyses, cells were incubated for 1 h at 37 °C in glucose- and galactose-free DMEM (Agilent Technologies) containing 5 nM ranirestat. ECAR analysis was conducted using the XF Glycolysis Stress Test (Agilent Technologies) by sequentially injecting glucose, galactose, oligomycin, and 2-deoxglucose at concentrations of 5.6 mM, 25 mM, 1 μM, and 50 mM, respectively.

### 4.10. Immunocytochemistry

Cover glasses harvested with IMS32 cells were fixed with 4% paraformaldehyde for 10 min and then blocked with Blocking One (Nakarai Tesque). After washing with PBS-T, target proteins were visualized using the following antibodies: mouse anti-TOMM40 monoclonal antibody (1:1000; Proteintech Group, Inc.), mouse anti-endoplasmic reticulum (ER)-resident Lys-Asp-Glu-Leu oligopeptide antigen (KDEL) monoclonal antibody (1/500, MBL, Nagoya, Japan), and chicken Alexa 488-conjugated anti-mouse IgG antibodies (1:400). These cells were mounted with Mounting Medium with DAPI-Aqueous, Fluoroshield (Abcam). Fluorescent images were obtained using a confocal laser scanning microscope (FV-1000, Olympus, Tokyo, Japan).

### 4.11. Statistical Analysis

All data are presented as the mean + standard deviation (SD). Statistical analyses were conducted using Easy R (EZR version 1.40) [57]. One-way analysis of variance (ANOVA) followed by post hoc Tukey HSD tests was applied for all data comparisons. *p*-values < 0.05 were considered statistically significant.

## Data Availability

All data presented in this paper are available in the manuscript or from the corresponding author (H.Y.).

## Supplementary Materials

The following supporting information can be downloaded at https://www.mdpi.com/article/10.3390/ijms26041529/s1.
