# Supplementary material for: Epalrestat Alleviates Reactive Oxygen Species and Endoplasmic Reticulum Stress by Maintaining Glycosylation in IMS32 Schwann Cells Under Exposure to Galactosemic Conditions
Source: Int J Mol Sci. 2025 Feb 12;26(4):1529. doi: 10.3390/ijms26041529 (PMC11855471; doi:10.3390/ijms26041529)
Supplement: Supplementary file 1 [file ijms-26-01529-s001.zip › ijms-3477808-supplementary.pdf]

Epalrestat attenuates reactive oxygen species and endoplasmic reticulum stress by maintaining glycosylation in Schwann cells under galactosemic conditions

Hideji Yako, Naoko Niimi, Shizuka Takaku, Junji Yamauchi, Kazunori Sango

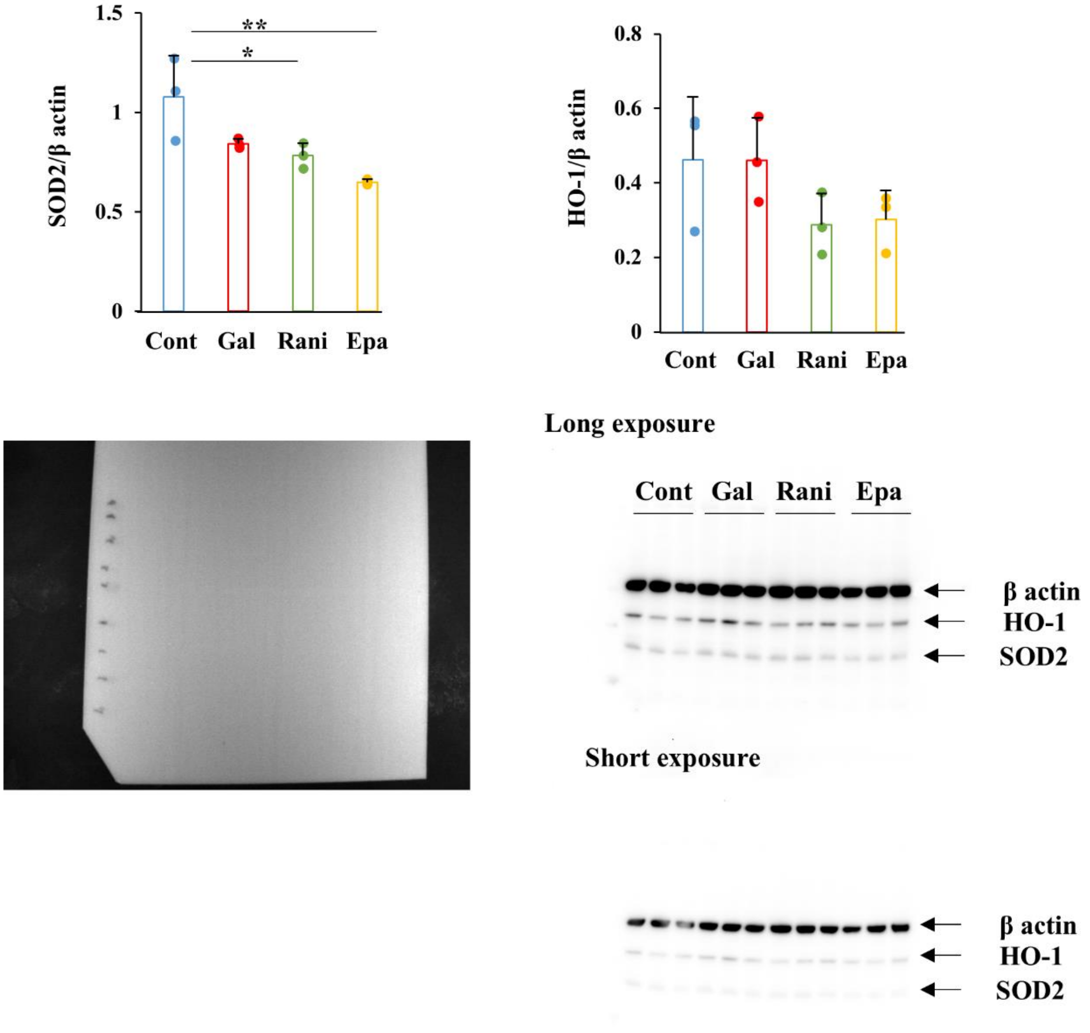

Figure S1. AR inhibitors did not change SOD2 and HO-1 expression.

The levels of SOD2 and HO-1 were determined using Western blotting. The bars show the levels of these proteins in IMS32 cells under control (Cont; **blue**), and high-galactose (Gal; **red**) conditions in the additive with 5 nM ranirestat (Rani; **green**) and epalrestat (Epa; **yellow**). Values represent mean + SD from three experiments (individual values are depicted as circles). * $P < 0.05$.

Full length of the membranes SOD2, HO-1, and β actin were visualized in the same membrane.

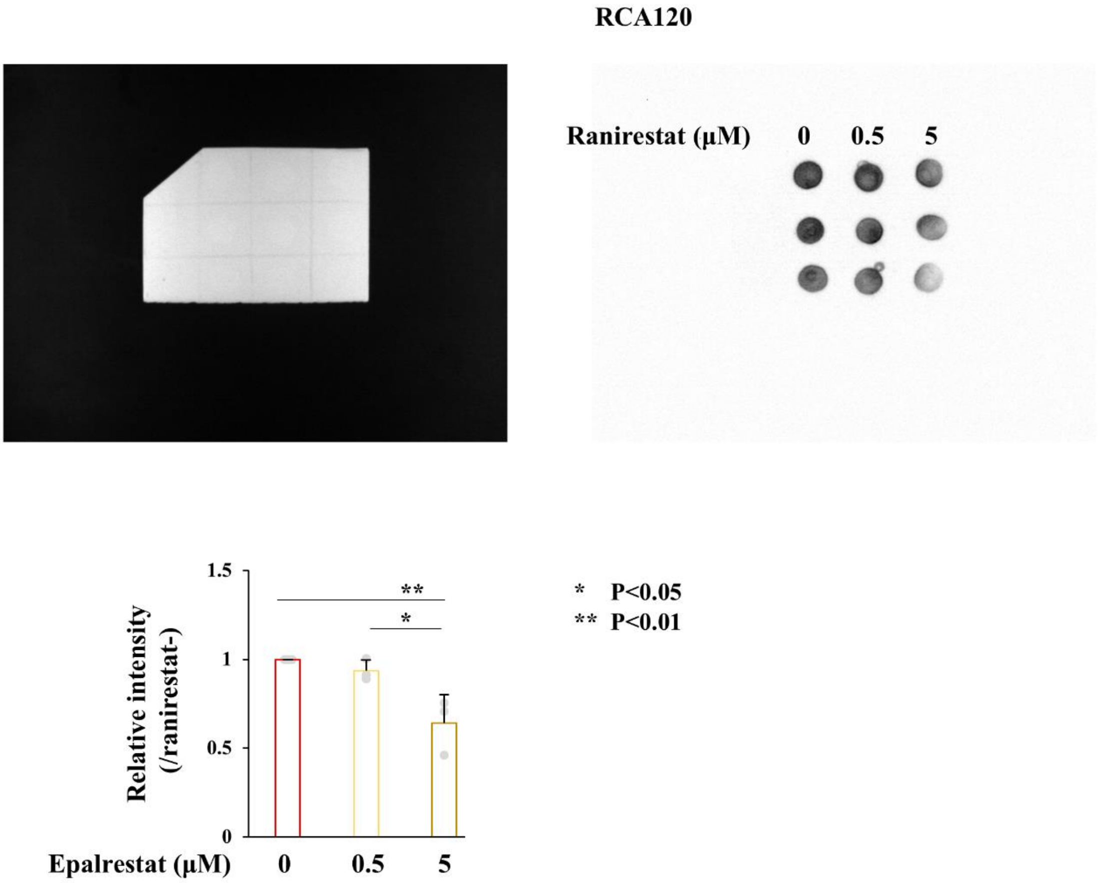

Figure S2. Epalrestat inhibited RCA120 expression in a dose-dependent manner.

The levels of RCA120 were determined using dot blot. The bars show the levels of RCA 120 in IMS32 cells under control high-galactose (**red**) conditions in the additive with 0.5 (**yellow**) and 5 μM epalrestat (**brown**). Values represent mean + SD from three experiments (individual values are depicted as circles). * $P < 0.05$ and **<0.01.

The actual membrane RCA120 were exhibited.

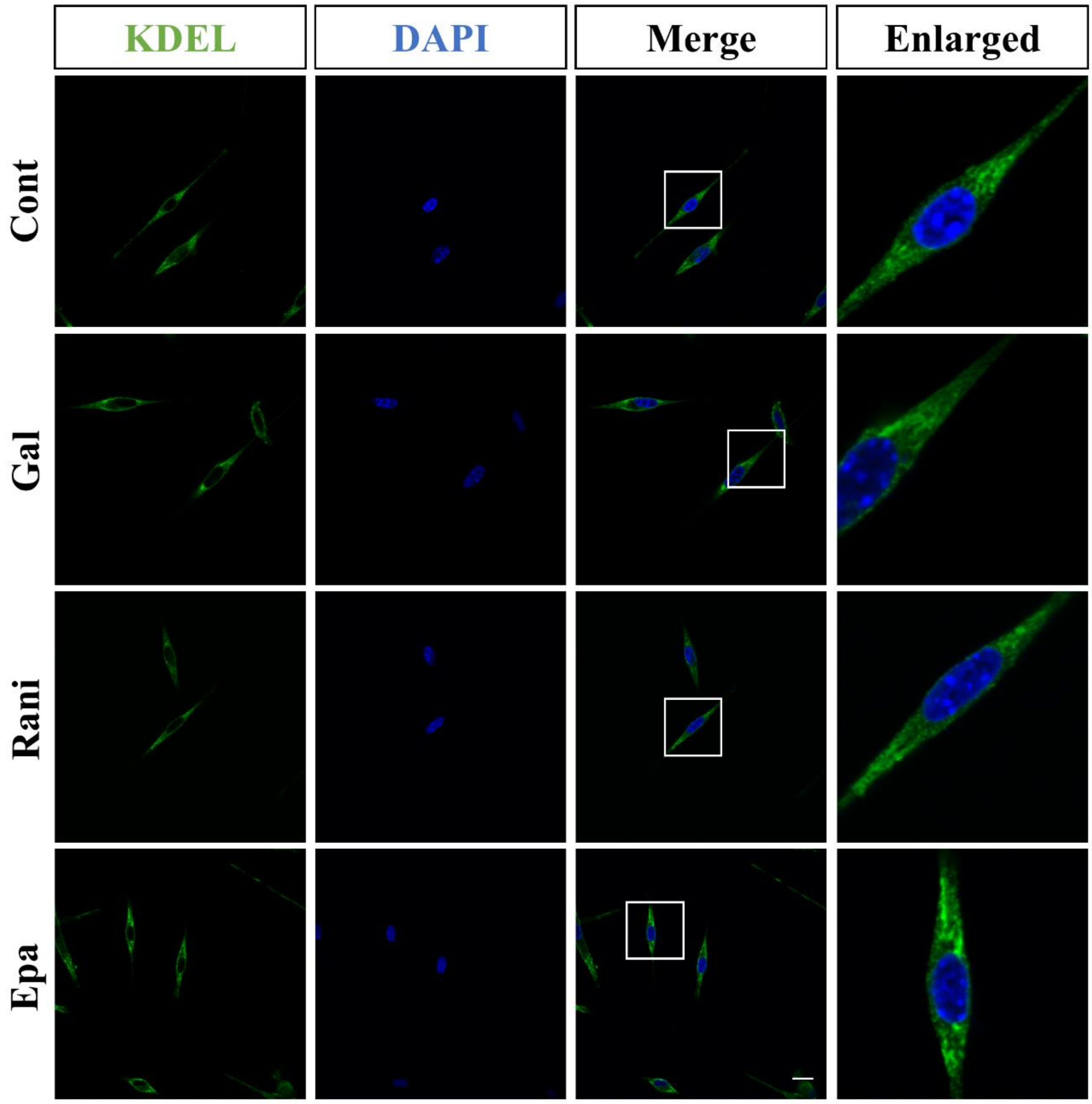

Figure S3. KDEL signals were not changed under high-galactose conditions.

Representative immunostaining images of the KDEL (**green**) and nuclear staining of DAPI (**blue**) in IMS32 cells under the following conditions are shown: control (Cont), high-galactose (Gal), high-galactose with 5 nM ranirestat (Rani), and high-galactose with 5 nM epalrestat (Epa). Enlarged images of the white-boxed regions in the merged images are also shown. Scale bar: 10 μm.

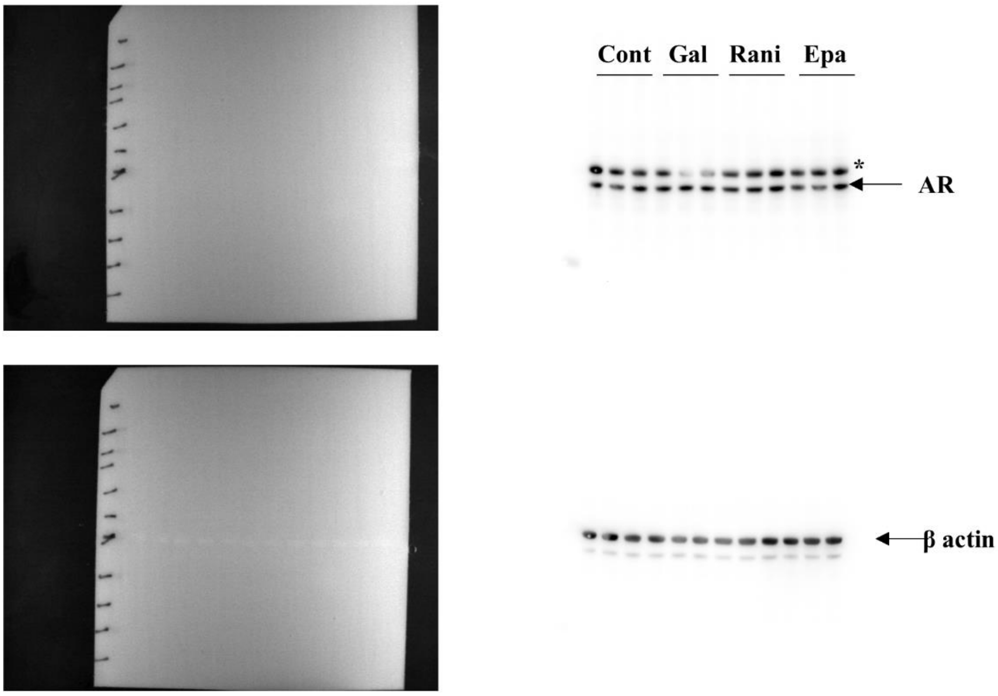

Figure S4. The actual Western blotting images of AR and β actin.

Full length of the membranes AR and β actin presented in **Fig. 2A** were sequentially visualized in the same membranes. * shows the bands of another antibody.

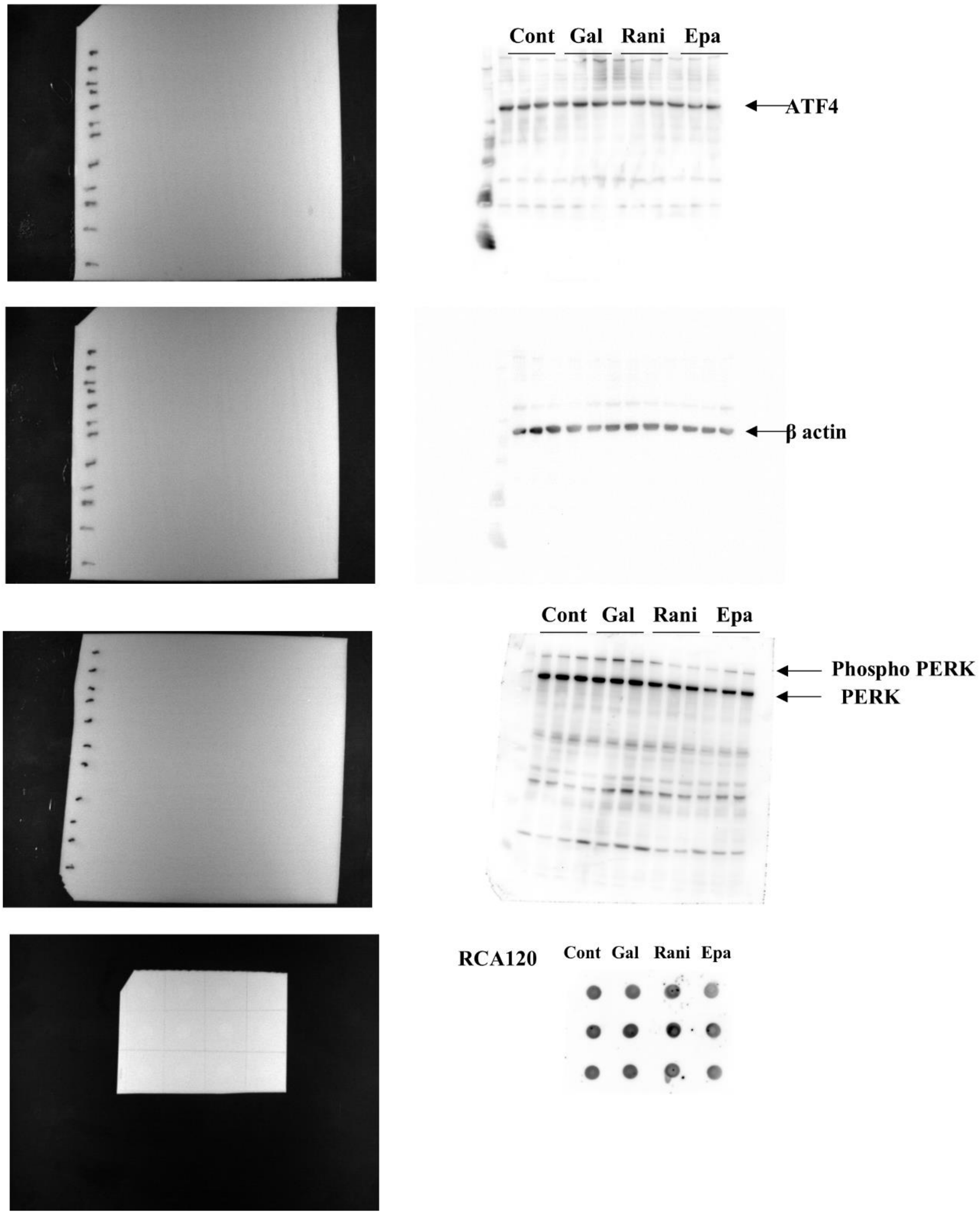

Figure S5. The actual Western blotting images of ATF4, β actin, Phospho PERK, PERK, and RCA120.

Full length of the membranes ATF4, β actin, Phospho PERK, PERK, and RCA120 presented in **Fig. 5** were sequentially visualized in the same membranes of ATF4 and β actin.
